# The Chemical Basis of Intracerebral Hemorrhage and Cell Toxicity With Contributions From Eryptosis and Ferroptosis

**DOI:** 10.3389/fncel.2020.603043

**Published:** 2020-12-08

**Authors:** Paul J. Derry, Anh Tran Tram Vo, Aswini Gnanansekaran, Joy Mitra, Anton V. Liopo, Muralidhar L. Hegde, Ah-Lim Tsai, James M. Tour, Thomas A. Kent

**Affiliations:** ^1^Center for Genomics and Precision Medicine, Department of Translational Medical Sciences, Institute of Biosciences and Technology, College of Medicine, Texas A&M Health Science Center, Houston, TX, United States; ^2^Department of Neurosurgery, Center for Neuroregeneration, The Houston Methodist Research Institute, Houston, TX, United States; ^3^Division of Hematology, Department of Internal Medicine, McGovern Medical School, University of Texas Health Science Center at Houston, Houston, TX, United States; ^4^Department of Chemistry, Rice University, Houston, TX, United States; ^5^Department of Computer Science, George R. Brown School of Engineering, Rice University, Houston, TX, United States; ^6^Department of Materials Science and NanoEngineering, George R. Brown School of Engineering, Rice University, Houston, TX, United States; ^7^Stanley H. Appel Department of Neurology, Institute for Academic Medicine, Houston Methodist Hospital, Houston, TX, United States

**Keywords:** intracerebral hemorrhage, ferroptosis, senescence, oxytosis, reactive oxygen species, iron, hemoglobin

## Abstract

Intracerebral hemorrhage (ICH) is a particularly devastating event both because of the direct injury from space-occupying blood to the sequelae of the brain exposed to free blood components from which it is normally protected. Not surprisingly, the usual metabolic and energy pathways are overwhelmed in this situation. In this review article, we detail the complexity of red blood cell degradation, the contribution of eryptosis leading to hemoglobin breakdown into its constituents, the participants in that process, and the points at which injury can be propagated such as elaboration of toxic radicals through the metabolism of the breakdown products. Two prominent products of this breakdown sequence, hemin, and iron, induce a variety of pathologies including free radical damage and DNA breakage, which appear to include events independent from typical oxidative DNA injury. As a result of this confluence of damaging elements, multiple pathways of injury, cell death, and survival are likely engaged including ferroptosis (which may be the same as oxytosis but viewed from a different perspective) and senescence, suggesting that targeting any single cause will likely not be a sufficient strategy to maximally improve outcome. Combination therapies in addition to safe methods to reduce blood burden should be pursued.

## Introduction

Intracerebral hemorrhage (ICH), contributing to 50% of all stroke morbidity and mortality, is an often devastating neurovascular disease without an effective therapy, affects more than 15 million people worldwide annually. Larger ICH and cerebral microbleeds are linked to ~1.8–2.4-fold higher risk of both acute and chronic neurological dysfunction, long-term disability, cardiovascular dysfunction, and increased predisposition to neurodegenerative disorders (Yates et al., 2014; Ghosh et al., 2015; Akoudad et al., 2016; Vijayan and Reddy, 2016; Kitago and Ratan, 2017; Vijayan et al., 2018a). A major challenge in the clinical management of ICH is the lack of mechanistic insight causing neuronal and vasculature toxicity. While inflammation, oxidative injury (reactive oxygen species, ROS) and excess redox-active iron appear to play critical roles in ICH-induced neurotoxicity, efforts at exploring individually antioxidant-or chelation-based therapeutic strategies have not proven effective in late-stage clinical trials (Perry et al., 2002; Margaill et al., 2005; Firuzi et al., 2011; Yeatts et al., 2013; Shirley et al., 2014; Duan et al., 2016; Fouda et al., 2017; Selim et al., 2019), thus warranting a deeper understanding of the mechanisms and pathways.

Definitive therapy for ICH remains elusive, with prognosis tied to the severity of the initial hemorrhage, the extent of rebleeding, and longer-term deleterious effects of blood and its breakdown products (Xi et al., 2014; Vijayan and Reddy, 2016; Sidyakin et al., 2018). In addition to the initial injury, processes associated with brain injury during ICH include red blood cell (RBC) lysis, which results in the release of free hemoglobin (Hb; Augustynek et al., 2014; Dang et al., 2017). Excess free Hb is toxic to neurons, the endothelium, and vasculature (Balla et al., 1991; Jeney et al., 2002; Zille et al., 2017). Though the precise time course of RBC lysis after clinical or experimental ICH is variable, as much as 10 mM hemin is liberated from Hb in the peri-ICH region (Qureshi et al., 2005; Chen-Roetling and Regan, 2017).

Currently, no therapy has definitively improved outcomes from ICH. Recently, 1-year outcomes following treatment with the chelator, deferoxamine, appeared to favor this therapy, but primary outcomes measures did not show improvement (Yeatts et al., 2013; Selim et al., 2019), and so the ultimate benefit remains ambiguous. Analysis of outcomes following removal of blood using catheter-based therapy plus fibrinolytics (MISTIE-III) showed some promise if blood removal reached a certain threshold (Hanley et al., 2019). In our view, this later therapy, with the goal of early removal of blood non-surgically following ICH, has considerable promise given how toxic the hemorrhage is, although multiple questions remain as to the optimum approach and the extent of benefit remains to be seen.

In this review article, we will discuss potential mechanisms of injury following the release of Hb and its breakdown products, mechanisms that suggest no single mode of therapy will be sufficient to minimize the deleterious effects that shape poor outcomes from this condition.

### Intracerebral Hemorrhage

Intracerebral hemorrhage (ICH) is caused by the rupture of blood vessels within the brain. Typical sites of bleeding include subcortical territory in the anterior and posterior regions, often a consequence of vascular changes following chronic hypertension, or cortical regions more typically due to vascular lesions associated with a variety of conditions. ICH causes mechanical damage through mass effect as a primary effect (Lai et al., 2014), excitotoxicity, and oxidative stress among many secondary effects (Xi et al., 2006). ICH has two classifications: primary, and secondary. Primary, or spontaneous ICH, makes up the majority of intracerebral hemorrhage cases (Fewel et al., 2003). The second classification, secondary ICH, is caused by an instigating event such as the hemorrhagic transformation of an ischemic stroke or traumatic brain injury involving a hemorrhagic contusion (Lok et al., 2011). Of the spontaneous cases, approximately 70% can be attributed to vessel fissure caused by hypertension (Wilkinson et al., 2018). Hypertension is associated with atherosclerosis in larger vessels and arteriosclerosis in smaller arterioles (Wityk and Caplan, 1992), with pathologically hyaline, thicker and more brittle, walls that are weaker than healthy arterioles (Fewel et al., 2003).

Water is a largely incompressible liquid and makes up most of the blood volume. Following a breach of the vessel wall, blood begins to pool and apply pressure to the tissue (mass effect of extravasated blood; Wilkinson et al., 2018). Expansion of the bleed can cause a characteristic deflection of the interhemispheric fissure (midline) and potentially bleed into the ventricles. These gross effects lead to microscopic damage in the form of neuronal and astrocytic network disruption caused by the stretching and tearing of axons as the tissue is compressed. Distortion of the tissue can compress vessels and cause localized ischemia, however there is still debate about this phenomenon since not all patients with ICH have a perihematomal penumbra (Aguilar and Brott, 2011). The perihematomal region is associated with a vasogenic form of edema, the buildup of fluid within the tissue (Urday et al., 2015; Grunwald et al., 2017; Lim-Hing and Rincon, 2017). Edema is a complex process but is mediated primarily by injury and molecular events within components of the blood-brain barrier (Xi et al., 2002; Simard et al., 2007; Lim-Hing and Rincon, 2017; Vaibhav et al., 2018).

The pooled blood is not only a source of pressure on the brain but is also a source of constituents usually confined to vessels, including the whole range of blood cells and plasma. One of the unique consequences of hemorrhage is the release of excess iron, a prooxidant (Wu et al., 2003). Red blood cells (RBCs) carry Hb, an oxygen trafficking protein that carries four heme prosthetic groups amounting to four atoms of iron. In a normal individual there is 30–44 mM iron in blood found as Hb, and other sources (Chernecky and Berger, 2012). The large amount of iron present in blood poses an oxidative threat to the surrounding tissue (Dixon and Stockwell, 2014; Mitra et al., 2014). RBCs pool in the hematoma and over time degrade, releasing Hb into the wound. No single mechanism is involved in this process. Macrophage and microglia-mediated cell death may also occur in the early stage of the hematoma. RBCs express CD47 on their surface as a “do not eat me” (Takimoto et al., 2019) signal where the absence or alteration of CD47 recruits their consumption (erythrophagocytosis) by macrophages (Hua et al., 2000; Lim-Hing and Rincon, 2017). Blocking CD47 has been shown to reduce hematoma size and increase the rate of its removal by promoting erythrophagocytosis by macrophages (Jing et al., 2019). There is also evidence of complement cascade activation and formation of Membrane Attack Complex (MAC) pores on RBCs, which cause the release of their intracellular components into the milieu (Hua et al., 2000; Cao et al., 2016).

In addition to CD47, another scavenger protein CD36, a differentially regulated microglial surface marker, is involved in the activation of microglia-mediated innate immune responses in several inflammatory neuropathological conditions like Alzheimer’s disease as well as in ICH (Febbraio et al., 2001; El Khoury et al., 2003; Silverstein and Febbraio, 2009). Elevated CD36 expression in the perihematomal region is well-correlated to experimental ICH outcomes in rodent models. Conversely, CD36 deficits in ICH patients have been associated with a slower hematoma clearance rate along with aggravated neuropathological conditions, compared to patients with normal CD36 expression. Reduced expression of CD36 is associated with increased production of pro-inflammatory M1-microglia mediators like TNF-α and IL-1β, thereby, inhibiting microglia phagocytosis as well as hematoma clearance (Fang et al., 2014). Another critical regulator of the inflammatory cascade is the Toll-like receptor 4 (TLR4) protein. TLR4 acts as the negative modulator of CD36 expression in microglia, and increased TLR4 expression results in poor recovery of ICH patients as well as slower absorption of hematoma in autologous blood-induced ICH rodent model (Fang et al., 2014; Lan et al., 2017a), TLR4 inhibitor TAK-242 has been shown to upregulate CD36 expression accelerating erythrophagocytosis of hematoma cells and suppressing H_2_O_2_ content in and around the ICH lesions (Fang et al., 2014) Furthermore, ICH-derived heme activates TLR4/MyD88 signaling-mediated upregulation of proinflammatory markers including IL-6 (Lin et al., 2012), which in turn leads to STAT3 phosphorylation, ultimately inducing the expression of hepcidin, a master regulator of iron metabolism, which is associated with chronic cognitive impairment in ICH survivors (Wrighting and Andrews, 2006; Xiong et al., 2016).

Increased hepcidin levels have been observed in both serum and brain after ICH induction. Serum hepcidin inhibits brain iron efflux from microvascular endothelial cells and macrophages by binding to the iron exporting channel protein ferroportin. Moreover, an increase in hepcidin expression is found in various brain cells like astrocytes, microglia, and neurons following ICH, and considered a contributor to brain oxidative injury (Xiong et al., 2016). On the other hand, IL-6 stimulation acts as the potent inducer for NF-κB-mediated inflammatory signaling and its nuclear translocation (Wang et al., 2003). To prevent the extensive oxidative DNA damage by the resulting massive cytokine storms, nuclear factor erythroid 2-related factor (NRF2) binds to the antioxidant response element (ARE) DNA sequences thereby inhibiting inflammatory responses and increasing heme oxygenase-1 (HO-1) expression through activation of the antioxidant defense mechanism following ICH induction (Ma and He, 2012; Sivandzade et al., 2019).

The induction of NRF2 expression has been found well-correlated with improved blood-brain barrier integrity as well as motor and cognitive functions in several neurodegenerative disorders (Sivandzade et al., 2019). The feedback loop of the NRF2-p62 axis has is involved in RBC degradation through erythrophagocytosis in bone-marrow-derived macrophages, which could also act in a similar way to clean up hematoma cells after ICH (Santarino et al., 2017). Apart from the antioxidant machinery, complement activation contributes to another dimension of the host defense mechanism. The terminal effector of this mechanism, MAC, has been found to activate proinflammatory responses *via* induction of noncanonical NF-κB signaling in endothelial cells (Jane-Wit et al., 2015).

### Heme and Hemoglobin

The vast majority of the iron in blood comes from Hb with the remainder, 7.2–29 μM, from iron bound to serum proteins. Consequently, the primary source of damaging iron in ICH comes from the release of iron from RBCs. Iron is stored in the form of Hb, a covalently linked tetrameric peptide containing four heme moieties (Perutz, 1989). Hb is the primary carrier of oxygen in the body next to myoglobin, found in the muscles (Cotton et al., 1999; Alayash et al., 2001).

**Figure 1:**
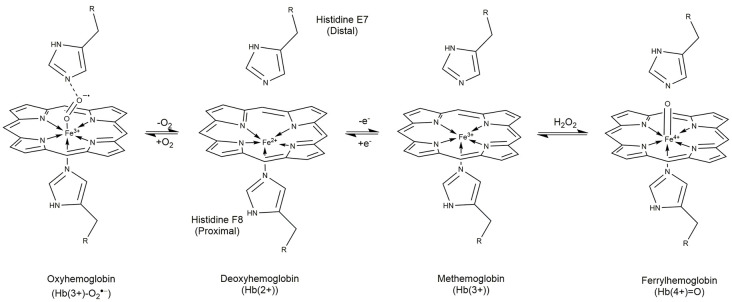
Structures and interconversion of oxygen in the prosthetic heme found in hemoglobin. Heme contains four subunits, each with an individual heme moiety. In oxyhemoglobin, Hb(3+)-O_2^-_ is predominant and is found in equilibrium with deoxyhemoglobin [Hb(2+)]. Loss of an electron gives Hb(3+) as found in methemoglobin. Oxidation of the Hb(3+) by hydrogen peroxide produces ferryl hemoglobin [Hb(4+) = O] which degrades to Fe(3+) and porphyrin degradation products. The oxidation state of hemoglobin and it’s extra/intracellular compartmentalization are effective biomarkers by MRI for ICH age.

Heme is found in the oxygen carrier hemoglobin as a prosthetic moiety. Hb carries four heme molecules, one in each of the four globin molecules that make up Hb. Each Hb molecule is composed of two αβ dimers (Perutz, 1989; Buehler et al., 2020). Heme B, the metalloporphyrin found in Hb, is made from protoporphyrin IX coordinated to a Fe(2+) cation (Figure 1). In Hb, heme is bound to each globin polypeptide through imidazolyl nitrogen on histidine F8. The Fe(2+) cation sits slightly below the plane due to its size (Perutz, 1989; Cotton et al., 1999). The heme is pulled from the center towards the histidine F8 at the center with an approximately 0.4 Å deflection (Perutz, 1989; Cotton et al., 1999; Figure 1). When bound to the proximal histidine, heme has one unoccupied axial position perpendicular to the plane of the protoporphyrin IX (Perutz, 1989; Cotton et al., 1999). In Hb, heme exists in the +2, +3, and +4, +5 (in the presence of hydrogen peroxide; Gumiero et al., 2011) oxidation states leading to different charge isomers of Hb. The precise charge of oxyhemoglobin has been a point of debate for many years with three competing theories. In this review, we will use the findings made by Weiss et al. and supported by later Mössbauer studies by Lang and Marshall and Sharrock et al. (Lang and Marshall, 1966; Sharrock et al., 1976; Shikama, 1998; Huang and Groves, 2018). In order of charge, hemoglobin can exist in the following states with the corresponding heme group: oxyhemoglobin [Hb(3+)-${\text{O}}_{2}^{•-}$, oxyferroheme], deoxyhemoglobin [Hb(2+), ferroheme], methemoglobin [metHb(3+), ferriheme], and ferrylhemoglobin [Hb(4+)=O, ferrylheme; Kasai et al., 2018; Figure 1]. Notably, the MRI T1 and T2 signal from pooled blood in the hematoma correlate to the different oxidation states of heme and its location inside and outside erythrocytes (Kidwell and Wintermark, 2008; Dang et al., 2017; Liu et al., 2019).

Canonically, Hb transports oxygen in red blood cells *via* ferroheme. Dioxygen binds reversibly to the Fe(2+) center of ferroheme to form oxyferroheme (oxyhemoglobin) at an angle 60° from normal through its sp^2^-hybridized molecular orbital (Figure 1). Due to the electronegativity of the oxygen, electron density from the Fe(2+) is pulled to the dioxygen, the reduction in electron density planarizes the oxyferroheme from its original puckered conformation according to Weiss (1964); Perutz (1989) and Shikama (1998). Histidine E7 above the opposite face, distal to the oxyferroheme binds to the dioxygen through its imidazolyl ε–nitrogen, at a complementary 120° angle making a chain to the oxyferroheme below (Figure 1; Perutz, 1989; Shikama, 1998; Cotton et al., 1999).

In addition to reversibly binding dioxygen, oxyhemoglobin can autoxidize to form methemoglobin and superoxide (Weiss, 1964). Methemoglobin can undergo a second transformation whereupon histidine E7 binds to the unoccupied axial site of the ferriheme to form a molecule called hemichrome (Rifkind et al., 1994; Riccio et al., 2002). This state may be a normal transition state In this conformation dioxygen is unable to bind to the iron since the axial positions are occupied by histidine (Riccio et al., 2002). However, the bis-histidine isomer is reversible unlike some isomers containing cysteine coordinated to the iron which eventually precipitates as Heinz bodies, or insoluble aggregates of hemichrome found in erythrocytes towards the end of their lives (Blumberg and Peisach, 1971; Winterbourn and Carrell, 1974; Rifkind et al., 1994). The formation of hemichromes may also occur by the attack of the Fe(2+) by the distal imidazolyl nitrogen in oxyferroheme, which displaces dioxygen as superoxide and oxidizes ferroheme to ferriheme (Rifkind et al., 1994).

### Hemoglobin Redox Cycling

**Figure 2:**
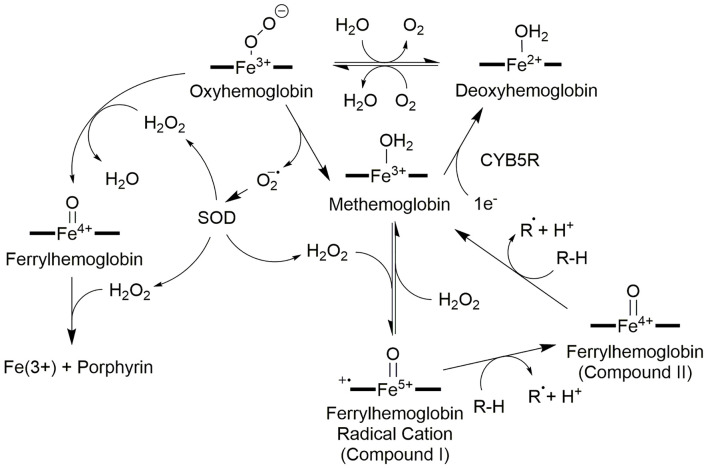
Redox network of hemoglobin oxidation states. The center of the hemoglobin redox network is methemoglobin which can be reduced or oxidized to two intermediate products: deoxyhemoglobin *via* cytochrome b5 reductase (CYB5R), or ferrylhemoglobin radical cation with H_2_O_2_, respectively. Oxygenation of deoxyhemoglobin forms oxyhemoglobin, and the introduction of a reducing agent such as an unsaturated fatty acid to ferrylhemoglobin radical cation (Compound I) produces ferrylhemoglobin (Compound II). Oxyhemoglobin can also become ferrylhemoglobin if H_2_O_2_ is present; a second H_2_O_2_ destroys the porphyrin. Ferrylhemoglobin may also oxidize a second reduced species to return to methemoglobin. Oxyhemoglobin also plays a role in generating the reactive oxygen species needed for these oxidation state changes: oxyhemoglobin may spontaneously disproportionate to liberate superoxide which is converted to H_2_O_2_ by superoxide dismutase (SOD).

Oxyhemoglobin and methemoglobin are part of a reduction-oxidation cycle driven by autooxidation of oxyhemoglobin (Figure 2) followed by catalytic reduction from NADH:cytochrome b5 oxidoreductase (Misra and Fridovich, 1972; Wallace et al., 1982; Balagopalakrishna et al., 1996; Nagababu and Rifkind, 2000). The autooxidation of oxyhemoglobin produces superoxide, a process studied by EPR by Balagopalakrishna et al. (1996), and methemoglobin (Misra and Fridovich, 1972; Wallace et al., 1982; Nagababu and Rifkind, 2000; Kasai et al., 2018). Superoxide may also be produced by monotopic ligand (CN^−^, Cl^−^, or others) displacement of dioxygen from oxyhemoglobin or reduction in pH (Wallace et al., 1982). Superoxide production has the knock-on effect of producing H_2_O_2_ either through self-dismutation or catalytic dismutation by superoxide dismutase (Rifkind and Nagababu, 2013). Hemoglobin salvage by NADH:cytochrome b5 oxidoreductase (CYB5R) was discovered by Passon and Hultquist (1972); methemoglobin can be reduced back to hemoglobin completing the cycle (Hultquist and Passon, 1971; Figure 2). Congenital methemoglobinemia is associated with a deficit in NADH:cytochrome b5 reductase and presents as impairment of the hemoglobin salvage pathway and inefficient oxygen transport by erythrocytes (Ashurst and Wasson, 2011).

The H_2_O_2_ produced by the dismutation of superoxide drives two additional pathways centered upon oxyhemoglobin. In red blood cells, there is a steady-state concentration of approximately 2 × 10^−10^ M H_2_O_2_ at any given time (Nagababu and Rifkind, 2000). If Oxyhemoglobin reacts with H_2_O_2_, a ferrylhemoglobin [Hb(4+)=O] species is generated and upon the introduction of a second H_2_O_2_ produces methemoglobin and superoxide (Figure 2). Excess superoxide may destroy the porphyrin and release the iron (Misra and Fridovich, 1972; Giulivi et al., 1994).

On the other hand, approximately 3% of the Hb in a healthy erythrocyte autoxidizes in 24 h and likely generates the majority of the hydrogen peroxide at baseline (Giulivi et al., 1994). The resulting methemoglobin can react with the H_2_O_2_ produced from superoxide to form a ferrylhemoglobin radical cation [Fe(4+)=OPP^•+^; Compound I; Nagababu and Rifkind, 2000]. The radical is delocalized across the porphyrin as in most heme-containing oxygen-activating enzymes (Nagababu and Rifkind, 2000). In the ferryl state (Compound II), the iron oxidation state is +4 and a single oxo ligand is bound to the open axial position [Fe(4+)=O]. The ferrylhemoglobin radical is a single electron oxidizer and has at least two substrates of interest: hydrogen peroxide and unsaturated lipids (Nagababu and Rifkind, 2000). Hydrogen peroxide can donate 2e^−^ to the ferrylheme radical cation to produce ferriheme and water in a peroxidase-like reaction (Alayash et al., 2001). Second, ferrylheme radical cation can abstract a hydrogen atom from unsaturated lipids to produce lipid peroxides (Chutvanichkul et al., 2018) and Fe(4+)=O. In a second reaction, Fe(4+)=O abstracts an electron from a second lipid or other reducing substance to generate methemoglobin and a second radical (Nagababu and Rifkind, 2000; Potor et al., 2013). Because superoxide and H_2_O_2_ are produced in red blood cells (Giulivi et al., 1994; Alayash et al., 2001), it is quite likely that Fenton and Haber–Weiss reactions are predominately driving the formation of lipid peroxides and cellular damage as opposed to direct oxidation by hemoglobin (Ohgami et al., 2005).

### Release and Capture of Iron in Intracerebral Hemorrhage

Eryptosis, a programmed suicidal death pathway of red blood cells (Foller et al., 2008), is a component of intracerebral hemorrhage (Dang et al., 2017; Liu et al., 2019). Eryptosis occurs for reasons such as oxidative stress, inhospitable osmolarity, and changes in membrane composition (Foller et al., 2008) including the appearance of phosphatidylserine phagocytosis markers on the outer leaflet of the membrane (Foller and Lang, 2020). The death of a red blood cell triggers the release of its contents into the milieu. The potential concentration of iron-containing detritus can be substantial with approximately 10 mM in iron or hemin (Robinson et al., 2009). The clearance of the iron takes place *via* several mechanisms. First, blood products (methemoglobin, hemin, and iron cations) can be released by eryptosis and the uncontrolled cell death pathway, hemolysis (Foller et al., 2008; Dang et al., 2017; Buehler et al., 2020). Second, damaged red blood cells can be consumed by macrophages through erythrophagocytosis (Bulters et al., 2018).

Methemoglobin decays in the blood into two αβ subunit dimers. Haptoglobin, a dimeric (trimeric, or tetrameric; depending on which exons are transcribed) polypeptide is expressed by hepatocytes to bind hemoglobin αβ subunit dimers in circulation. Adult humans have a wide range of circulating haptoglobin levels (0.3–1.9 mg/ml) which binds the hemoglobin subunit with effective irreversibility at a rate constant of 5.5 × 10^5^ M^−1^s^−1^ (Kristiansen et al., 2001; Buehler et al., 2009, 2020). Haptoglobin has the additional function of reducing the reactivity of hemin to oxygen while in circulation (Mollan et al., 2014). Mollan et al. (2014) found that the Hp1–1, Hp2–1, and Hp2–2 phenotypes all interact with hemoglobin to prevent the loss of heme from hemoglobin dimers into the milieu. This effect reduces the rate of heme autoxidation but does not have a clear effect on ferryl heme formation (Mollan et al., 2014) meaning that the protective capacity of haptoglobin may lay mostly in its ability to remove hemoglobin from the blood and not necessarily to arrest ROS generation.

After haptoglobin binds to two αβ dimers it binds to the macrophage-expressed multidomain transmembrane receptor CD163 (Kristiansen et al., 2001). In addition to macrophages, the expression of CD163 has been reported in hippocampal neurons following experimental hemorrhagic stroke (Garton et al., 2017b; Liu et al., 2017). This finding suggests that neurons may be inappropriately responding to the locally higher concentration of hemoglobin as they do not express heme oxygenase 1 (HO-1), the faster, and inducible of the HO-1/2 pair (Dore et al., 1999; Garton et al., 2017a).

The lack of expression of haptoglobin in the brain parenchyma and cerebrospinal fluid as opposed to serum is dramatic: the reference range for haptoglobin in serum is 0.3–1.9 mg/ml, however, Chang et al. (2013) reported that the concentration of cerebrospinal fluid haptoglobin in 22 healthy adults was 0.060 mg/dL (0.006 mg/ml; Chang et al., 2013). Likewise, Loeffler et al. (1999) reported the concentration of haptoglobin in the brain tissue of rats was on average (of all brain regions) 0.06 ng/mg (wet weight; Loeffler et al., 1999, p. 1710). Both astrocytes and oligodendrocytes have been shown by Lee et al. (2002) and Zhao et al. (2009), respectively, to be an inducible source of the majority of haptoglobin found in the brain. In rats, Zhao et al. (2009) showed that sulforaphane enhanced haptoglobin expression. Compared to control rats, oligodendrocyte expression of haptoglobin was upregulated by 10 times over the ICH-naïve control at day 1, and as much as 40 times at day 3 following intrastriatal injection of hemolyzed blood. Rats treated intraperitoneally with sulforaphane and lysed RBCs had a 30-fold more robust expression on day 1 than those treated with PBS. Serum expression rose and fell over the time window for both sulforaphane and PBS-treated rats with a maximum at day 1 (Zhao et al., 2009). The robust haptoglobin response to hemoglobin began shortly after the time of the modeled ICH and the response decreased over time.

The lack of haptoglobin in the CSF and brain parenchyma likely limit the acute buffering capacity of the central nervous system to hemoglobin which the circulatory system is otherwise able to handle. While upregulation within the brain may at some level keep up with the diffusion of the hemorrhage products, lack of resident cells to generate haptoglobin in the CSF may relate to the poorer prognosis that often accompanies ventricular extension, particularly when associated with other clinical factors including involvement of the 3rd and 4th ventricles (Daou et al., 2017).

Some implications of blood in the CSF can come from work with subarachnoid hemorrhage (Galea et al., 2012). The fate of blood and its breakdown products in the CSF is still unclear. In subarachnoid hemorrhage-naïve patients, CSF levels of CD163 are approximately 50-fold lower than in serum (Galea et al., 2012). Following SAH, CSF CD163 levels increase 8-fold. The cells responsible for producing CD163, macrophages, and microglia, are likely entering the subarachnoid space, and as Galea et al. (2012) reported, not all of the hemoglobin in CSF following SAH is captured by haptoglobin, most hemoglobin is found free in the CSF despite the presence of haptoglobin. Passive or other mechanisms may be involved in the clearance of hemoglobin from the CSF (Rennels et al., 1985; Galea et al., 2012; Iliff et al., 2012). Another trap for blood products is hemopexin which binds heme and is both expressed intrathecally and imported into the CSF from the circulation (Garland et al., 2016), discussed further below.

The combined findings suggest several key points. Lee et al. demonstrated that CD163 levels increased in the CSF after ICH likely due to the infiltration of macrophages and microglia (Lee et al., 2002). Chang et al. (2013) note that CSF hemoglobin levels fall even in the absence of a robust haptoglobin response which suggests that hemoglobin can be passively removed from the CSF as long as the removal system is functional. Haptoglobin administration may be a potential therapy to reduce the detrimental effects of excessive hemoglobin in the nervous system following ICH (Garland et al., 2020). Ultimately it seems that the absorption capacity of hemoglobin in the brain is inducible to an extent, and that haptoglobin on its own is not necessarily the sole participant. There may be translational avenues that point to using haptoglobin as a treatment for hemorrhagic stroke; work by Zhao et al. (2009) showed that sulforaphane upregulates haptoglobin expression and Hugelshofer et al. (2019) showed that direct injection of haptoglobin into the CSF reduced vasospasm and the diffusion of hemoglobin into the brain parenchyma from the CSF in sheep.

**Figure 3:**
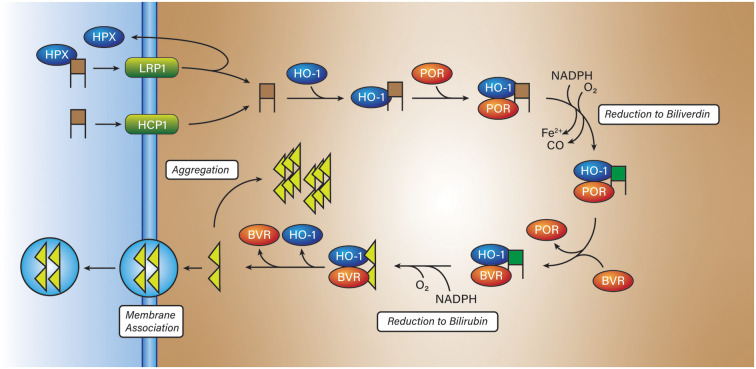
Hemin breakdown. Hemin (brown squares) is imported into most cells *via* either low-density lipoprotein receptor-related protein (LRP1) or proton-coupled folate transporter (HCP1). In the former state, hemin is transported into the cytosol bound to hemopexin (HPX). In the latter case, hemin is directly transported into the cytosol. Once inside the cytosol, hemin is reduced to biliverdin (green squares) by NADPH:cytochrome P450 reductase (POR) and then bilirubin (yellow triangles) by biliverdin reductase (BVR). The product, bilirubin, has two fates, aggregation within the cell, or aggregation into the membrane with the possibility of entering the circulatory system.

Hemin is poorly soluble in water and is often carried by a protein in the solution including but not limited to albumin or the heme-trapping protein hemopexin (Robinson et al., 2009; Figure 3). Hemopexin has a very high affinity for hemin (*k*_d_ = 10^−13^ M) and can remove hemin from the blood (Robinson et al., 2009; Chen-Roetling et al., 2012), or due to its high affinity for heme, remove it directly from (met)hemoglobin, and may act as an antioxidant (Miller et al., 1996). Hemopexin has a singular receptor, LRP1 (low-density lipoprotein receptor-related protein; CD91) expressed on a multitude of cell types and found throughout the body (Moestrup et al., 1992; Figure 3). Upon binding to LRP1, the hemopexin-hemin complex is endocytosed, the hemin undocks, and the hemopexin is transported back across the membrane into circulation. Other than protein-mediated transport, hemin is also known to be taken up through the proton-coupled folate transporter (PCFT/HCP1) receptor expressed in astrocytes and neurons directly into the cytosol (Robinson et al., 2009; Dang et al., 2010, 2011; Figure 3). Haptoglobin and hemopexin have different fates *in vivo*, haptoglobin, with its Hb-αβ dimer is degraded by macrophages whereas hemopexin with its lone heme molecule is recycled back into circulation (Smith and Mcculloh, 2015).

### Heme Breakdown Products

Hemin retains its oxidizing characteristics when abstracted from hemoglobin and if consumed by a cell needs to be degraded to prevent a runaway oxidation cascade. To render hemin a lesser threat, cells express heme oxygenase 1 or 2 (HO) to degrade hemin or other intracellularly produced hemes, to biliverdin, ferrous iron, and carbon monoxide. Heme oxygenase 2 (HO-2) is expressed continuously in neurons and is not inducible (Chang et al., 2003). The regulation mechanism of HO-2 is not clear, however recent work by Liu et al. (2020) demonstrated that HO-2 is stabilized by hemin. HO-2 carries three docking sites for heme but only one is catalytic, the remaining two are heme regulatory motifs that bind heme; unlike other heme-regulated proteins, HO-2 is only destabilized by a loss of heme binding to the catalytic site, not the regulatory sites (Liu et al., 2020). Heme oxygenase 1 (HO-1) is not expressed in the brain under normal circumstances but is expressed in macrophages (Naito et al., 2014; Vijayan et al., 2018b), microglia (Schallner et al., 2015), and astrocytes localized to the peripheral tissue surrounding the hematoma from ICH (Chang et al., 2003; Yu et al., 2016). Some evidence by Yu et al. (2016) suggests that HO-1 is expressed in neurons under oxidizing conditions elicited by 1-methyl-4-phenylpyridinium (MPP+) or in spinal cord neurons following injury (Lin et al., 2016). However, HO-1 induction was shown by Nitti et al. (2018) to not occur in neurons in cortical tissue exposed to traumatic injury out to 30 days. Conversely, Yu et al. (2016) reported that HO-1 expression in neurons does not change until after 24 h and lags the immediate expression by astrocytes. HO-1 and HO-2 are membrane-bound through a C-terminal hydrophobic tail and are found most commonly on microsomal membranes (Unno et al., 2007). HO-1 and HO-2 are isozymes with only 43% amino acid sequence homology (Unno et al., 2007). Heme oxygenases work in tandem with NADPH:cytochrome P450 reductase (POR) and biliverdin reductase (BVR) to degrade hemin to bilirubin, ferrous iron, and carbon monoxide (Unno et al., 2007; Munoz-Sanchez and Chanez-Cardenas, 2014; Figure 3). Work by Wang and De Montellano (2003) showed that cytochrome P450 reductase binds to HO-1 with a binding affinity (*k*_d_ = 0.4 ± 0.1 μM). On the contrary, Spencer et al. found a *k*_d_ of 16.4 μM for HO-2 with POR. However, Spencer et al. also found that using their surface plasmon resonance method, that the *k*_d_ for HO-1 with POR was 20.4 μM, that may reflect differences in technique (Spencer et al., 2014).

In the catalytic site of heme oxygenase, several steps must take place to break open the protoporphyrin ring, release CO, ferrous iron, and bilirubin. First, hemin enters the binding pocket and coordinates to His25 an arrangement not dissimilar to the coordination pocket in hemoglobin. In the first reaction step, hemin [Fe(3+)] is reduced to heme [Fe(2+)] by the transfer of a single electron from P450 reductase. Next, dioxygen enters the binding pocket and coordinates to the heme. The addition of a second electron reduces the oxo-heme to hydroperoxo-heme. The next product, α-meso-hydroxyheme, is a hydroxylated product with an -OH on the alpha carbon and is formed through a single step. The nature of this single step is unclear as it may involve a concerted proton-driven reaction or the formation of Compound I [Fe(4+)=OPP^•+^] or Compound II [Fe(4+)=OPP] as part of the hydroxylation step (Unno et al., 2007). The conversion from α-meso-hydroxyheme to verdoheme is a multistep process initiated by dioxygen followed by the release of CO, reduction of the ferric iron to ferrous iron by P450 reductase, and placement of cationic bridging oxygen at the alpha position (Matsui et al., 2010). Verdoheme is oxidized to biliverdin by dioxygen, and four electrons provided by P450 reductase. The product is a porphyrin ring cleaved at the alpha position where the carbons adjacent to the bridging oxygen are now ketones. The ferrous iron leaves the cleaved ring and enters the Labile Iron Pool (LIP). In total, the conversion of hemin to biliverdin requires seven electrons donated from the NADPH-dependent cytochrome P450 reductase and three dioxygen molecules, the HO-P450 reductase complex catalyzes the production of one equivalent of CO, Fe(2+), and biliverdin.

The conversion of biliverdin to bilirubin also occurs in the binding pocket of HO by biliverdin reductase. Biliverdin reductase is NADPH-dependent and binds to HO-1 in humans and may compete for the heme-binding pocket that cytochrome P450 reductase also binds (Wang and De Montellano, 2003). Competition for the active site is ultimately a consequence of both reductases participating in the degradation of hemin while still requiring the oxidase activity that heme oxygenase can provide. The main function of biliverdin reductase in the conversion of hemin to bilirubin, and is the reduction of the methine carbon to methylene. This action produces the flexible bilirubin product and is released upon the undocking of biliverdin reductase. In rats, bilirubin does not undergo mono- or diglucuronidation in the brain by uridine diphosphate-glucuronosyl transferases and therefore is not made more water-soluble (Suleman et al., 1993). It is worth noting that defective glucuronidation in the liver by UGT1A1 is associated with neurotoxicity and hyperbilirubinemia (Ouzzine et al., 2014).

Evidence for neuroprotection and neurotoxicity by bilirubin is mixed. Work by Dore et al. (1999) showed that rat hippocampal and cortical neuron cultures treated with PKC-inducer phorbol-12-myristyl-13-acetate (PMA) were protected against H_2_O_2_-mediated toxicity. In this model, PMA induced protein kinase C (PKC) to phosphorylate HO-2. In doing so HO-2 was stimulated producing more bilirubin. Bilirubin was detectable by a substrate-selective antibody, and to show that the effect was due to HO-2, tin protoporphyrin IX was used to inhibit HO-2. Rat cultures expressing HO-2 showed no toxicity against 60 or 80 μM H_2_O_2_ when treated with 0.1 μM PMA; note that a reduction in viable cells was reported with 1 μM PMA, an effect suggested to be due to downregulation of PKC (Dore et al., 1999). The authors reported that bilirubin is more protective when bound to human serum albumin than on its own and only at concentrations higher than 100 nM does it show toxicity on its own (Dore et al., 1999).

These findings were conducted with low concentrations of bilirubin (< 250 nM), the standard total circulating blood bilirubin concentration in humans is between 1.71 and 20.5 μM and therefore may not necessarily reflect the reported findings *in vivo*. Bilirubin diffuses into, and out of the brain across the blood-brain barrier if it is not glucuronidated (Bratlid, 1990; Figure 3). Bilirubin can also reside within the lipid bilayers either through its carboxylic acid moieties that can bind with quaternary ammonium groups on phosphatidylcholines or through hydrophobic binding in the core of the lipid bilayer (Bratlid, 1990; Figure 3).

Despite the potential neuroprotective aspects of bilirubin, bilirubin may also contribute to the edema associated with ICH. Loftspring et al. (2010) hypothesized that unconjugated bilirubin (UBR, indirect bilirubin) such as that produced by injured cortical tissue, stimulates the release of cytokines to recruit microglia, macrophages, and neutrophils to the injury. It was later shown that bilirubin increased neutrophil infiltration of mice injected with autologous whole blood (Loftspring et al., 2011). Fluorescence microscopy of frozen sections showed a marked increase in the neutrophil count after treatment with bilirubin compared to untreated control ~90 vs. ~40 (*n* = 4–5; *p* ≤ 0.05) as measured by a count of Ly-6G positive cells and a ~30% reduction (*n* = 4–5, *p* ≤ 0.05) in macrophage or microglial cells as measured by a count of F4/80 positive cells. The introduction of neutrophils earlier to the injury may be associated with a heightened inflammatory response and comorbid edema (Clark et al., 2008; Loftspring et al., 2011). Other potential toxic effectors are the oxidation products of bilirubin (Pyne-Geithman et al., 2005). Bilirubin, while insoluble in water, is avidly bound by plasma proteins and can be transported to the liver (Clark et al., 2008; Ritter et al., 2016).

### The Fate of Ferrous Iron

Ferric iron has a reduction potential of +0.770 V (1e^−^ + Fe^3+^/Fe^2+^) and can reduce dioxygen (−0.330 V; 1e^−^ + O_2_/${\text{O}}_{2}^{•-}$) to superoxide or H_2_O_2_ to hydroxyl radical (+0.380 V; H^+^ + 1e^−^ + H_2_O_2_/HO• + H_2_O). Dioxygen reduced by Fe(2+) to superoxide can be dismutated by SOD to H_2_O_2_, O_2_ and H_2_O. The resulting H_2_O_2_ can be reduced by Fe(2+) through the Fenton reaction to hydroxyl radical and hydroxide (Fe^2+^ + H_2_O_2_ -> Fe^3+^ + HO• + HO^−^). In a third reaction, ${\text{O}}_{2}^{•-}$ can reduce Fe(3+) to Fe(2+) whereupon H_2_O_2_ can be reduced to HO•, O_2_, and HO^−^ through the Haber-Weiss reaction. Hydroxyl radical rapidly peroxidizes proteins (albumin: 8 × 10^10^ M^−1^ s^−1^), nucleic acids (RNA: 1 × 10^9^, DNA: 8 × 10^8^ M^−1^ s^−1^), and lipids (linoleic acid: 9 × 10^9^ M^−1^ s^−1^) as well as the antioxidants ascorbate and glutathione, 1 × 10^10^ and 1.4 × 10^10^ M^−1^ s^−1^, respectively (Davies, 2016).

A third reaction, superoxide produced by dioxygen reacting with Fe(2+) can also react with H_2_O_2_ to produce HO•, O_2_, and ^−^OH through the Haber–Weiss reaction.

Ultimately, all roads lead to hydroxyl radicals if iron is involved. The intracellular check mechanism of free iron is its capture and storage by ferritin (FTH; Harrison and Arosio, 1996). Ferritin stores iron in the form of a superparamagnetic ferrihydrite core that stores approximately 4,500 iron atoms (Harrison et al., 1976; Chasteen and Harrison, 1999). Ferritin is a spontaneously self-assembled spherical protein cage consisting of 24 subunits with two functional elements, a cage for the de-mineralization of the biomineralized iron, and the transport into and out of the cage *via* a 3-fold channel (Badu-Boateng and Naftalin, 2019). The regulation of iron transport through the channel depends on the availability of ascorbate and hydrogen peroxide to reduce and oxidize iron as it leaves and enters the ferritin, respectively. H_2_O_2_ and ascorbate compete for the reduction site of ferric iron leading to a regulatory effect: under a reducing environment, iron is released into the cytosol, however, under an oxidizing environment, iron is sequestered in the ferritin (Badu-Boateng and Naftalin, 2019).

Another check on ferrous iron is lactoferrin (LTF), a serum iron transport protein produced by neutrophils analogous to transferrin (Ohgami et al., 2005; Zhao et al., 2018). Lactoferrin tightly binds ferric iron (*k_d_* = 10^−20^ M) and prevents its participation in the Fenton cycle with ferrous iron (Zhao et al., 2018). The key difference between transferrin and lactoferrin is that lactoferrin is less sensitive to pH than is transferrin and can maintain the chelation of its iron payload (Baker and Baker, 2004; Zhao et al., 2018). While transferrin loses its iron at pH = 6, lactoferrin can maintain its iron chelation until pH = 5 (Baker and Baker, 2004). In an experimental mouse model of ICH using autologous blood injections, Zhao et al. (2018) found that LTF levels in the brain increased despite no change in its mRNA levels. Neutralization of circulating neutrophils using the Ly-6G antibody caused significant degradation of neurological deficit scores (NDS; ~5.9 to ~6.8, *n* = 10, *p* < 0.05) 3 days after the injection. *In vitro* cell models indicated that adding lactoferrin to media containing lysed RBCs was efficacious in a concentration range of 10–1000 μg/ml at reducing cytotoxicity in cocultured neuronal and glial cells (*n* = 3, *p* < 0.05; Zhao et al., 2018). Further support to the potential beneficial effects of lactoferrin is a study by Zhao et al. (2020) in which injection of a modified lactoferrin derivative with a longer half-life reduced NDS after injection in several different ICH models even with a 24 h window.

### Mechanism of Ferroptosis

**Figure 4:**
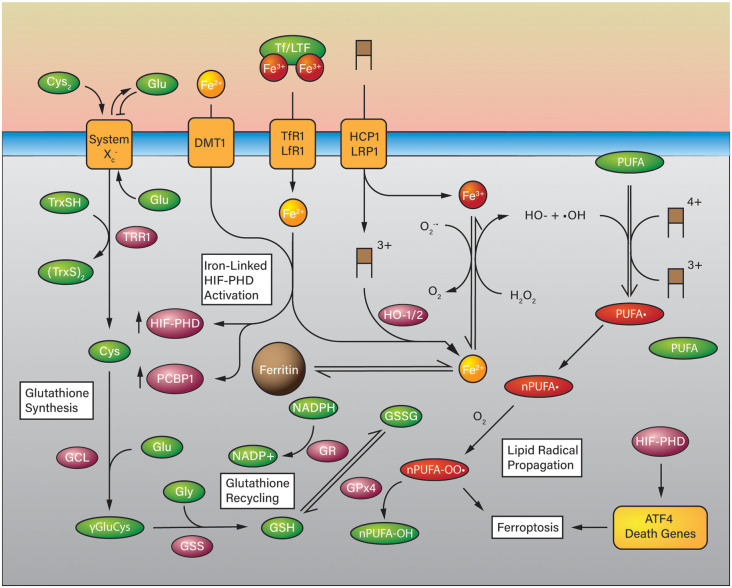
Ferroptosis in the Context of Iron-Overload in ICH. Glutathione is synthesized using cystine as a substrate for cysteine which is coupled to glutamate and glycine in two steps. Glutathione can be used as a reducing substrate for glutathione peroxidase (GPX4) to reduce polyunsaturated fatty acid (PUFA) lipid peroxides to PUFA-OHs and is oxidized to glutathione disulfide (GSSG). GSH can be recycled from GSSG by glutathione reductase at the expense of NADPH. In a high iron-overload situation such as ICH, iron is imported into the cell in a heme group (brown boxes) or otherwise *via* divalent metal transporter 1 (DMT1), transferrin receptor (TfR1), low-density lipoprotein receptor-related protein (LRP1), and proton-coupled folate transporter (HCP1). Heme degradation takes place *via* HO-1 or HO-2 (HO-1/2) which releases Fe(2+) into the cell that can participate in the Fenton reaction. Unregulated ferrous iron in the cell can react with hydrogen peroxide to form hydroxyl radicals which can generate PUFA radicals and eventually PUFA peroxides in the presence of oxygen which are quenched by GPx4. Iron overload may also activate HIF-PHDs and the expression of ATF4 death-associated genes.

Ferroptosis appears to be a form of cell death that can be triggered by glutathione (GSH) depletion and iron excess which leads to the accumulation of lipid reactive oxygen species (L-ROS; Cao and Dixon, 2016; Figure 4). Cystine (Cys_2_) is imported *via* the cystine/glutamate antiporter System ${\text{x}}_{c}^{-}$ (SLC7A11) and is used to synthesize the stoichiometric antioxidant GSH (Lewerenz et al., 2013). GSH is synthesized through a multistep process. Cystine from the extracellular space is exchanged for glutamate and then reduced by intracellular glutathione, or thioredoxin reductase 1 (TRR1) to cysteine (Cys) and CyS-SG (-SG; glutathiyl; Conrad and Sato, 2012). Once reduced, cysteine is conjugated to glutamate *via* its side chain carboxylate by glutamylcysteine ligase (GCL) to form γ-glutamylcysteine. Glutathione synthetase (GSS) conjugates glycine to the cysteinyl carboxylate to form glutathione as the product (Doll and Conrad, 2017). As discussed, glutathione can act on its own to reduce oxidized species, or it can act as a substrate for enzymes such as glutathione peroxidase 4 (GPX4; Cao and Dixon, 2016; Ingold et al., 2018). GPX4 is the principal regulator of polyunsaturated fatty acid (PUFA) oxidation (Feng and Stockwell, 2018). GSH depletion or inactivation of GPX4 results in the uncontrolled oxidation of PUFAs to form PUFA peroxyl radicals (PUFA-OO*) and PUFA peroxides (PUFA-OOH; Doll and Conrad, 2017), which then are anchored to the plasma membrane by ACSL4 and LPCAT3 (Doll et al., 2017; Agmon et al., 2018), leading to membrane damage and ferroptosis sensitization (Cao and Dixon, 2016).

Lipid peroxides are the critical intermediate that activates ferroptosis by oxidizing PUFAs in lipid membranes and forming additional PUFA peroxyl radicals. This chain reaction is normally inhibited by GPX4 by a two electron reduction of PUFA-OOHs to non-toxic lipid alcohols (PUFA-OH; Girotti, 1998; Cao and Dixon, 2016). Arachidonic phosphatidylethanolamines, a specific class of PUFA, appear to mediate ferroptosis (Doll et al., 2017; Kagan et al., 2017; Agmon et al., 2018), suggesting that targeting the PE pathway provides a therapeutic approach to prevent cell death.

Ferroptosis can be triggered not only by GSH depletion but also by iron overload, e.g. hemoglobin degradation. At any given time there is an amount of iron available in the cytosol to be used elsewhere; this ill-defined feature is the Labile Iron Pool (LIP) and acts as a steady-state repository for iron as it is shuffled between demands (Chutvanichkul et al., 2018; Philpott et al., 2020). The composition of the LIP is not clearly defined, no specific set of molecules have been found associated with intracellular iron. However, there are known iron chaperones which transport iron specifically to different recipients (Hider and Kong, 2011; Philpott et al., 2020). Multiple mechanisms actively control iron homeostasis *via* FTH, transferrin (Tf), transferrin receptor (TfR), LRP1, HCP1, and ferroportin (FPN; Yan and Zhang, 2019). Tf and TfR import non-heme Fe^3+^ into the cell, where Fe^3+^ is reduced to Fe^2+^ by ferrireductases. Once absorbed, iron can be used for heme synthesis, exported *via* FPN, or incorporated into storage molecules FTH, thus, prevent iron retention and toxicity (Richardson and Ponka, 1997; Lei et al., 2019). Excess uptake and deficient storage result in an abundant Fe^3+^ ion in the cytoplasm. As Fe^3+^ accumulates and reaches the threshold Fe^3+^: Fe^2+^ ratio, from 1:1–7:1, iron produces massive ROS and initiates rapid lipid peroxidation *via* Fenton reaction (Braughler et al., 1986; Lesnefsky, 1992; Harrison and Arosio, 1996).

In a related pathway, cell death may be caused by excess iron being captured by chaperone PCBP1 which activates heat-inducible factor 1α prolyl hydroxylases (HIF-PHDs) that then translocate to the nucleus and promote the expression of death genes associated with leucine zipper transcription factor ATF4 (Chac1, chop, Trib3; Ratan, 2019). Indeed, multiple transcription factors are associated with ferroptosis (Dai et al., 2020), one of the most important is NFE2L2 or NRF2, a basic leucine zipper transcription factor that binds to antioxidant response elements (AREs) and thus plays a major role in regulating antioxidant genes as well as regulation of multiple anti-ferroptosis genes involved in iron metabolism (Ma, 2013; Shimada et al., 2016).

GPX4 is considered a central regulator of ferroptosis and a common therapeutic strategy is to address GPX4 and its downstream targets. Bersuker et al. (2019), Doll et al. (2019) and Kraft et al. (2020) demonstrate two GPX4-independent systems that modulate ferroptosis resistance. Bersuker et al. (2019) and Doll et al. (2019) showed that an endogenous ferroptosis suppressor protein (FSP1) acting parallel to the canonical GPX4. FSP1 is an NADH-dependent oxidoreductase. FSP1 then localizes to lipid droplets and the plasma membrane, reduces CoQ_10_, and suppresses lipid peroxidation. Disrupting the FSP1 pathway *via* pharmacological treatment with iFSP1 *in vitro*, and FSP1 knockouts *in vivo* sensitize the organism to ferroptosis (Bersuker et al., 2019; Doll et al., 2019). Recently, another pathway that regulates ferroptosis *via* CoQ_10_ was reported (Kraft et al., 2020). This pathway involved GTP cyclohydrolase 1 (GCH1), an enzyme required for the synthesis of CoQ_10_, and is upregulated in response to multiple ferroptosis inducers without altering GXP4-dependent factors. GCH1 selectively inhibits oxidation of di-polyunsaturated fatty acid phospholipids (Kraft et al., 2020) promoting resistance to ferroptosis and cell survival. GCH1 downstream molecules, especially dihydrobiopterin (BH2) and tetrahydrobiopterin (BH4) disrupt CoQ_10_ synthesis (Kraft et al., 2020) and suggest additional treatment targets (e.g., FSP1-CoQ_10_ and GCH1-BH4).

### Iron and the Development of Diseases

Because iron plays a vital role in biological processes, including oxygen transport, protein synthesis, and electron transport, it requires intensive regulation to maintain homeostasis. Relatively small changes can exhibit larger effects on the body, and has been implicated in disorders ranging from anemia (Abbaspour et al., 2014) to neurodegenerative diseases (Perry et al., 2018), reproductive disorders (Ng et al., 2019), cardiovascular disease (Kobayashi et al., 2018) and tumors (Basuli et al., 2017; Yamaguchi et al., 2018).

### Iron and Ferroptosis in ICH

ICH is an example of the pathological release of Hb and its derivatives. RBC lysis begins one day after the damage (Aronowski and Zhao, 2011), results in a sustainable iron buildup starting at day 3 to at least 4 weeks (Wu et al., 2003). Most of the iron distributes centrally in the basal ganglia, thalami, and white matter (Dietrich and Bradley, 1988). Nonheme iron not only generates excessive ROS and induces oxidative brain damage but appears to also activate ferroptosis and contributes to pathogenesis after ICH. As early as the 6 h time-point there is a 5.5-fold increase in Tf protein expression; seven days later Tf levels return to the basal level (Xie et al., 2019).

The use of ferroptosis inhibitors prevents neuronal death and can rescue neuron loss in a variety of models (Li et al., 2017a; Kenny et al., 2019; Xie et al., 2019). Significantly, ferrostatin-1, lipid radical-trapping antioxidant, at the most effective dose 10 μM *in vitro*, reduces Hb-induced cell death from 83 to 2% and decreases up to 50% lipid ROS formation (Li et al., 2017a). Other drugs targeting lipid ROS, such as the flavone triacsin C and the polyunsaturated hydrazone baicalein, also provide similar protections (Kenny et al., 2019) as inhibitors of the ferroptosis promoters 15-lipoxygenase 2 (15-LOX) and acyl-CoA synthetase long-chain family member 4 (ACSL4). Additionally, emerging studies have shown promising therapeutic benefits of ferroptosis inhibitors in the prevention of secondary brain injury caused by iron-mediated toxicity in rodent and piglet models (Hua et al., 2006; Gu et al., 2009; Okauchi et al., 2009; Jaremko et al., 2010; Li et al., 2017b). For example, post-ICH, iron accumulates, and is associated with caudate atrophy after 3 months (Hua et al., 2006). The administration of an iron chelator deferoxamine (DFO) reduced 50% of ICH-induced ferritin upregulation in the perihematomal zone (Hua et al., 2006). DFO also prevented neuronal degeneration and white matter injury in piglet models (Gu et al., 2009). A more brain permeable iron chelator, VK28, showed greater restoration of neurological function, white matter damage, and mortality rate than DFO (Li et al., 2017b).

Ferroptosis can also be inhibited with selenium. Alim et al. (2019) found that glutathione peroxidase 4 (GPX4) among other selenoproteins was upregulated following collagenase-induced ICH in rats. Also, hemin on its own can cause GPX4 expression to increase despite not rescuing the cell. Intracerebroventricular injection of the Se-containing Tat SelPep peptide reduced infarct volume after ICH (Alim et al., 2019). This finding is similar to that reported by Dharmalingam et al. (2020) with the nano-antioxidant PEG-HCCs, which sensitized to ferroptosis in murine endothelial cells while a deferoxamine-functionalized counterpart, DEF-PEG-HCCs reversed this effect (Dharmalingam et al., 2020).

### Roles of Macrophages and Microglia in ICH

The relationship between erythrophagocytosis and neuronal ferroptosis after ICH could be a promising clue to the pathogenic mechanism(s) driving the chronic neuropathological and neurodegenerative conditions in ICH. However, there is very limited information available so far on this mechanistic link. A recent study by Li et al. (2018) demonstrated brain ultrastructural alterations due to neuronal death and white matter injury following ICH in the collagenase rodent model. Notably, the study illustrated the co-existence of ferroptosis, autophagy, and necrosis in and around the ICH lesions. Axonal degeneration was observed in the acute phase of ICH, axonal demyelination was noticed in the striatum and corpus callosum in the subacute phase. Furthermore, rapid accumulation of activated resident microglia and infiltrating monocyte-macrophages was observed around the RBCs in the microvascular structures and degenerating neurons leading to erythrophagocytosis as well as clearance of neuronal debris from the lesions or hematoma regions. While protection against neuronal ferroptosis may be a key step to prevent overall brain injury in collagenase-induced ICH mice model (Li et al., 2017a), for erythrophagocytosis to occur in this setting, sequential activation and polarization of the microglial M1–2 phases require extensive cross-talks between various cytokines induction and neurons, astrocytes, oligodendrocytes and T lymphocytes (Lan et al., 2017b), providing multiple opportunities for intervention.

Efficient clearance of damaged RBCs and ferroptotic neurons are features of initiation of regeneration processes (Neumann et al., 2009; Youssef et al., 2018) Notably, excessive engulfment of damaged RBCs (erythrophagocytosis) surrounding the ICH lesions could, in turn, induce ferroptosis in monocyte-macrophages (Youssef et al., 2018), resulting in further neuronal damage, oxidative injury, and activation of proinflammatory responses. Studies have shown that inducible nitric oxide synthase (iNOS) expression and nitroxyl free radical enrichment of activated M1 microglia/macrophages essentially regulate their susceptibility toward ferroptotic death *via* reprogramming of lipid redox mechanism (Kapralov et al., 2020). Emerging studies have highlighted the pathogenic roles of ferroptosis in several most common neurodegenerative diseases as well as in ICH (Weiland et al., 2019).

### Iron and Brain Hypoxia in Hemorrhage

In ICH, insufficient oxygen delivery to the brain a hypoxic environment is induced, activating adaptation responses mediated by hypoxia-inducible factor (HIF). HIF 1-alpha (HIF-1α) has a key role in neuroprotection during hypoxia. Under normal conditions, HIF-1α is degraded by enzymes prolyl hydroxylase domain (PHD) or factor inhibiting HIF (FIH; Tian et al., 2011). The lack of oxygen causes PHD and FIH to be inactivated leading to HIF-1α upregulation and dimerization with HIF-1β to initiate transcription of hypoxia-responsive genes (Aragones et al., 2009).

Besides oxygen, iron is another necessary co-factor for PHD and FIH activities. Hence, iron chelators can be used to mimic hypoxia, activate HIF-1α, and inhibit neural injury in experimental models. For example, deferoxamine (DFO) can stabilize HIF-1α, and restore viability against oxidative stress (Siddiq et al., 2005). A similar compound, deferasirox (DFR) has advantages over DFO because it can be administered orally at half the dose than DFO with comparable neuroprotection within a 24 h onset (Zhao and Rempe, 2011). However, in clinical trials, any benefit has been modest, perhaps because of an overwhelming cascade of not just iron but other toxic breakdown products and/or that other cellular pathways are engaged, such as senescence, discussed below.

### Ferroptosis and Oxytosis May be the Same Pathway

Decades before the description of ferroptosis with its dependence on iron, a cell death pathway that resulted in similar endpoints including lipoxygenase activation and glutathione depletion was described, and because it occurred in the context of the release of reactive oxygen species was termed oxytosis (Murphy et al., 1989). The experimental conditions that led to the identification of oxytosis were exposure to the excitatory transmitter glutamate and depletion of glutathione, with a massive influx of calcium considered a major contributor (Murphy et al., 1989; Maher et al., 2018). Indeed, lipophilic antioxidants such as Vitamin E were able to mitigate toxicity (Miyamoto et al., 1989), the toxicity that was enhanced in low cystine (precursor to glutathione) media. The link to glutamate-stimulated inhibition of cystine transport was quickly made identifying glutathione depletion as a key event. As can be gathered from both the inciting events and consequences, oxytosis shares many similarities with ferroptosis. The key differentiator of these two cell death pathways was the more recent finding that intracellular iron was also increased, associated with alterations in transferrin receptor and ferritin chains, and the reduction in cell death that occurred with iron-chelating agents (Yang and Stockwell, 2008). Reviewing the overlap between these two pathways, some have concluded that indeed these represent a similar cell death process, with the later inclusion of iron as the primary differentiator (Lewerenz et al., 2018).

### Iron and Senescence in ICH

A variety of stress stimuli can, instead of causing cell death, induce a state of senescence (Childs et al., 2014, 2015). While usually associated with aging, cells can also undergo stress-induced premature senescence (SIPS; Sun et al., 2018) through events such as oxidative injury and acute DNA damage response signaling (Chen et al., 1995; Dharmalingam et al., 2020). Senescent cells are typically larger with altered organellar structure, characteristic expression of a variety of cell cycle arrest, inflammation, and other molecules (Yoon et al., 2006; Childs et al., 2015), including the accumulation in lysosomes of senescent associated beta-galactosidase (Lee et al., 2006). While not specific, a pattern of expression and cellular morphology can identify the transitional states, resulting ultimately in what is termed the senescence-associated secretory phenotype where cells secrete high levels of inflammatory mediators and other factors (Coppe et al., 2010). This phenotype can influence and indeed recruit neighboring cells.

Senescent cells also accumulate iron. However, iron overload in senescence does not sensitize the cells to ferroptosis. Instead, they inactivate ferritinophagy, the process of ferritin (FTH) degradation, and inhibit ferroptosis (Masaldan et al., 2018). Senescent cells enter the growth arrest phase and are resistant to a variety of cell death pathways, including ferroptosis. The hypothesis is supported if we carefully examine data from the downstream effect of GSH depletion. Besides lipid peroxidation and ferroptosis, it has been shown to simultaneously trigger autophagy and stress-induced premature senescence (SIPS; Sun et al., 2018), with SIPS potentially having a more dominant effect on cell phenotype. Under a variety of stressors that induce SIPS, cells survive and enter senescence instead of cell death, but the senescent phenotype can result in a nidus of inflammation, and resulting nidus for inflammation may induce further, non-ferroptotic mechanisms of injury contributing to cellular dysfunction (Dharmalingam et al., 2020).

Our group has recently linked senescence and ferroptosis in ICH models. Hemin (5 μM), likely through rapid induction of persistent DNA double strand breaks induced senescence in 40% of cultured neurons or endothelial cells after 24 h and prevented cell death after the addition of a normally toxic level (90 μM) of iron to the media (Dharmalingam et al., 2020). If the same occurred *in-vivo*, it would suggest that a subset of cells exposed to hemin become both pathological inflammatory *and* resistant to the high iron and oxidative stress environment generated in the ICH. While a highly active nano-antioxidant, PEGylated hydrophilic carbon clusters (PEG-HCCs) could reduce the presence of senescent cells, it increased sensitivity to ferroptosis and other cell death pathways after the addition of iron, thus in some ways negating the benefit of reducing senescent cells especially in the high iron-overload conditions and ROS milieu of ICH. Importantly, both senescence and ferroptosis could be prevented by treatment with deferoxamine-conjugated PEG-HCCs (Dharmalingam et al., 2020). This result underscores that addressing any single disease pathway may not be sufficient to ameliorate the major deleterious effects of ICH.

Because the accumulation of senescent cells is a feature of aging, their presence may influence outcome following ICH. It is generally thought that outcome is related to the survival of tissue, and in the context of ICH, cells are likely exposed to pathological processes for a prolonged period, and indeed, most recovery occurs within 6 months after the injury (Kitago and Ratan, 2017). Thus, cells must adapt to a persistent pathological and highly oxidating environment. In aging cells, iron accumulates and favors cells with iron-induced ROS resistance to survive (Toyokuni et al., 2020). Increased iron content is a feature of senescent cells and so senescent cells may be in a favorable condition to endure chronic ROS. However, given that senescent cells contribute to pathological inflammation, their ability to survive this chronic oxidative environment may inhibit good functional outcomes and may be a factor in poorer response from ICH related to age (Mandava et al., 2019).

## Conclusion

This review article has described the complex biology of Hb oxygen binding, the role of iron in regulating Hb function, and the multiple neurotoxic events associated with the release of Hb and its degradation products in ICH, all of which provide multiple opportunities to generate pathological processes. Cell death pathways have been described, including their vulnerabilities to intervention. Prominent among these targets include reactive radicals that are generated both as part of the redox reactions these constituents participate in but also potentiated through the release of free iron and its catalytic generation of additional radicals. While targeting some of these pathologies has been effective in pre-clinical models, the benefit has been underwhelming clinically. Removal of blood itself is a rational target but is invasive and the optimum methods and blood removal goals are still under investigation. Lactoferrin is emerging as a pleiotropic therapy with both excellent brain penetration, the capture of free iron, and multiple other potentially beneficial effects that await clinical confirmation (Zhao et al., 2018).

For the most part, however, non-iron mediated effects of blood breakdown products have been less of a focus, and prominent among these include hemin’s stimulation of both ferroptosis and the senescent phenotype. The resistance to ferroptosis by senescent cells had been well known and more recently confirmed after exposure to hemin, suggesting anti-ferroptosis therapy will be partially effective only in those cells not senescent. While the contribution of senescent cells to clinical outcomes in ICH is not established, in other conditions, it is considered a major factor in inflammation and downstream damaging effects. The upshot of these multiple events is that no single therapy is likely to be optimally effective, and therefore combination therapies, removal of blood plus therapies directed at specific cell death and phenotypic pathways may be required to yield therapies that can improve outcome from this devastating condition.

## Author Contributions

PD drafted manuscript and illustrated figures. AV and AG drafted manuscript. AL drafted manuscript and provided subject matter expertise. MH, A-LT and JT revised manuscript and provided subject matter expertise. TK, principal investigator, conceived of the concept, revised manuscript and provided subject matter expertise. All authors contributed to the article and approved the submitted version.

## Conflict of Interest

TK, PD, A-LT, JT, JM, and MH hold patents pending for the PEG-HCC and DEF-PEG-HCC particles described herein. The remaining authors declare that the research was conducted in the absence of any commercial or financial relationships that could be construed as a potential conflict of interest.
